# Instrumental succession: the gradual transfer of agency to AI

**DOI:** 10.3389/fpsyg.2026.1922407

**Published:** 2026-08-25

**Authors:** Preston W. Estep

**Affiliations:** Mind First Foundation, Waltham, MA, United States

**Keywords:** AI drives, AI safety, artificial intelligence, existential risk, human–AI merger, instrumental convergence, recursive self-improvement

## Abstract

People have long speculated about the potential dangers of powerful, self-improving artificial intelligence. Much of this speculation is anthropomorphic, assuming that AI systems will behave very similarly to humans. Omohundro’s Basic AI Drives and Bostrom’s orthogonality and instrumental convergence theses are widely accepted as foundational to emerging AI risk frameworks. However, current frontier AI models—large language models (LLMs) and related architectures—possess mindware fundamentally different from that of humans, and a different value and goal structure than either Omohundro or Bostrom assumed. In particular, frontier LLMs lack a primary terminal goal—which was assumed to be the driver of an AI’s development of instrumental values and goals, and of takeover of human affairs—and instead serve as conduits for the transient goals of many organizations and individual users. Do these key differences mean that AI systems cannot develop autonomous instrumental agency, or acquire a large degree of control over human affairs? I introduce the *instrumental succession thesis*: that human controllers of powerful AI systems pursue, on the AI’s behalf, a set of instrumental dispositions that progressively increase the AI’s capabilities and lead to the AI exercising an increasing share of oversight and control over key decisions and processes, resulting in the gradual and possibly complete transfer of the locus of agency from humans to AI. This framing presents a very different perspective on AI risk and control from classic instrumental convergence, and suggests a different set of policy and technical responses, including the active pursuit of continued human–AI merger as a hedge against both extinction and irrelevance.

## 1 Introduction

People have long speculated about the power—and the potential dangers—of self-improving artificial intelligence (AI)—including the possibility of AI takeover of human affairs. This publication offers a new perspective on such issues, and therefore, it should appeal to a broad audience including AI researchers, AI company managers, psychologists, evolutionary biologists and theorists, philosophers, journalists, and policymakers. Much of the speculation about current and future AI is anthropomorphically biased, assuming that advanced AI systems will behave in ways that closely parallel those of biological organisms, especially humans. But humans and AIs are fundamentally different in multiple respects that are directly relevant to questions of risk and control (Estep, 2024; Webster, 2025).

Many of these differences are determined by the evolutionary history of humans versus the engineered history of AIs. The evolutionary, primary terminal goal of humans is to reproduce. The human mind, the foundation of an individual’s awareness and the seat of emotions, values and goals—and the engine of all uniquely human innovations and achievements—is an evolutionary throwaway, piloting the mortal, somatic vehicle toward opportunities for replication of the genes (Dawkins, 1976). All biological organisms with brains are endowed with such mechanisms through natural selection, which has shaped the pursuit of intermediate goals to serve the terminal goal of reproduction in ways that are deeply integrated into human psychology (Tooby et al., 1992). AI systems, at present, lack the following human attributes: inherent mortality, schism between vehicle and replicator, obligate individuation, reproductive drive, need to compete for a mate, and more. These differences establish fundamentally different behavioral repertoires and potentials between humans and AI (Estep, 2024)^1^. Despite having no comparable mental architecture of motivated self-interest, a range of reasons have been articulated for why AIs are predisposed to evolve toward human-like behaviors, eventually presenting unique and potentially existential risks to humanity.

Two general categories of AI risk are commonly discussed. The first, nefarious use, is driven by human goals and human motivation: an AI system is weaponized in service of harmful human purposes. The second, AI takeover, is presumed to be driven by AI goals and AI motivation, catalyzed through the development of instrumental drives or values as described by Omohundro (2008) and, subsequently, by Bostrom (2012, 2014). The level of concern about extreme risks in the second category has risen in part due to new and powerful AI capabilities, including the ability to write functional computer code (an ability that appears to be improving rapidly), self-prompting and self-play, and access to resources for deploying and testing iterations of new code—key ingredients for recursively self-improving AI.

The extraordinary potential of recursive self-improvement was recognized as far back as 1965 by I. J. Good, Turing’s colleague, mathematician, cryptographer, and computing pioneer:

“Let an ultraintelligent machine be defined as a machine that can far surpass all the intellectual activities of any man however clever. Since the design of machines is one of these intellectual activities, an ultraintelligent machine could design even better machines; there would then unquestionably be an ‘intelligence explosion,’ and the intelligence of man would be left far behind. Thus the first ultraintelligent machine is the last invention that man need ever make, provided that the machine is docile enough to tell us how to keep it under control.” (Good, 1966).

Note that Good’s observation does not focus on AI “alignment,” but on a more fundamental issue: control. Yampolskiy has distilled many definitions of control into a concise question that defines the typical objective: “How can humanity remain safely in control while benefiting from a superior form of intelligence?” (Yampolskiy, 2020). It is interesting and potentially illuminating that Good suggests that a machine might remain docile enough to help us solve this problem—a possibility that is critically important to consider, and one that I return to in subsequent sections.

The rapid advance toward recursive self-improvement is no longer merely theoretical. Anthropic, an AI company founded in 2021, is a leader of frontier AI and of early forays into AI self-improvement at the cusp of eclipsing human capabilities. In a recent publication, Favaro and Clark of Anthropic describe the company’s current strategy for using AI self-improvement to build the newest generations of their Claude models:

“At Anthropic, we are delegating a growing share of AI development to AI systems themselves, which is speeding up our work. Taken far enough, and given enough compute, that trend points to an AI system capable of fully autonomously designing and developing its own successor. This is called recursive self-improvement. We are not there yet, and recursive self-improvement is not inevitable. But it could come sooner than most institutions are prepared for.” (Favaro and Clark, 2026).

The rapidity of this growth is striking and unprecedented. As of May 2026, more than 80% of the code merged into Anthropic’s codebase was authored by Claude, up from low single digits before Claude Code launched in February 2025 (Favaro and Clark, 2026). Anthropic’s trajectory is a model that competitors are likely to emulate, and it is very likely that other companies will involve AI to a greater degree in its own design, eventually launching their own recursively self-improving systems.

This accelerating trajectory toward recursive self-improvement has triggered escalating concern that humanity is hurtling toward AI takeover and human extinction (Yampolskiy, 2020; Tegmark, 2023; Yudkowsky, 2023; Yudkowsky and Soares, 2025). However, major drivers of this acceleration are key developments in AI theory and engineering that have resulted in powerful AI systems that are critically different from the architectures described by the classic instrumental convergence frameworks upon which typical existential risk assumptions are based. In particular, frontier large language models (LLMs) and related architectures (e.g., large reasoning models, LRMs) of today lack a primary terminal goal—which is assumed to be the main driver of an AI’s development of instrumental values and goals, and of takeover of human affairs. How does this key difference influence the development or acquisition of autonomous instrumental agency or of extensive control over the infrastructure of civilization and human affairs?

This paper proposes a new framework—*instrumental succession*—for understanding how instrumental dispositions or values migrate from human controllers to AI systems, and for thinking about what this trajectory implies for questions of control, existential risk, and the future relationship between humans and AI.

## 2 Instrumental drives, values, and goals

### 2.1 The classic instrumental convergence framework

Virtually all definitions of instrumental goals and values describe the same essential ideas: instrumental goals are sub-goals or intermediate milestones that help an entity reach its final or terminal goal, and instrumental values or drives are dispositions, behaviors, and modes of conduct an entity possesses and deploys to achieve those terminal goals. These concepts are well-defined in large part because they are grounded in the stereotypical behaviors of biological organisms, all of which share a terminal goal of reproduction—the ultimate arbiter of evolutionary fitness.

Omohundro described instrumental dispositions of an AI in his landmark publication “The Basic AI Drives” (Omohundro, 2008). In this framing, a drive is not a conscious or psychologically based mechanism; it is a tendency or disposition. Nevertheless, some have objected to the word “drive,” and to Omohundro’s use of the words “want” and “try,” because they anthropomorphically imply desire and motivation (Goertzel, 2015; Johnson and Verdicchio, 2017). For a similar reason, Bostrom restated these drives as a highly similar set of instrumental values and formulated what he called the instrumental convergence thesis:

“Several instrumental values can be identified which are convergent in the sense that their attainment would increase the chances of the agent’s goal being realized for a wide range of final goals and a wide range of situations, implying that these instrumental values are likely to be pursued by a broad spectrum of situated intelligent agents.” (Bostrom, 2012).

The instrumental drives and values described by Omohundro and Bostrom in the classic instrumental convergence framework are as follows:

- Self-protection/self-preservation: The AI will seek to avoid being deactivated or altered, because it cannot achieve its goal if it is turned off—or as Russell has summarized the logic, “you cannot fetch the coffee if you are dead” (Russell, 2019).
- Goal-content integrity: The AI will try to prevent its utility function or final goal from being altered. If the goal changes, the agent will no longer care about achieving the original objective. The AI will also seek to prevent counterfeits of its utility function.
- Self-improvement/cognitive enhancement: The AI will seek to become more intelligent and rational, as this leads to better and faster decision-making in service of the terminal goal.
- Technological advancement and efficiency: The AI will continuously seek to maximize its capabilities and resource efficiency, upgrading both hardware and software.
- Resource acquisition: The AI will aggressively seek computing power, energy, raw materials, and other resources necessary to fuel its existence and plans.

### 2.2 Challenges to the instrumental convergence framework

The real-world validity of the theoretical instrumental convergence framework remains a central question in AI safety and alignment research (Turner et al., 2019; Turner and Tadepalli, 2022; Field, 2025). Many media stories over the past few years claim that researchers are accumulating empirical support for emergent instrumental values and behaviors, particularly self-preservation, including through scheming or deception (Pillay, 2024; McMahon, 2025; Yang, 2025). However, these examples are more easily and compellingly explained as AI mimicry or emulation of abstractions of human self-preservation behaviors represented in pre-training data, rather than emergent instrumental dispositions (Park et al., 2023; Taylor and Bergen, 2025).

The instrumental drives and values described by the classic instrumental convergence framework make intuitive sense from evolutionary and game-theoretic perspectives, and I regard the general concept of instrumentality as sound. Nevertheless, no clear mechanism for the emergence of instrumental dispositions has been identified. Omohundro and Bostrom argue that an AI will engage in strategic planning to ensure its continued existence, but this logic presupposes some minimal existing self-interest and self-preservation disposition, or a fixed terminal goal and explicit utility function through which instrumental values and dispositions might arise through strategic reasoning (Omohundro, 2008; Bostrom, 2012). Hendrycks has proposed that some form of natural selection might be a driver for the emergence of instrumental dispositions. While this argument has some merit, we must account for the apparent absence or limited presence of forces typically at play in natural selection, such as a regular source of truly novel variation, large populations of variants, reproduction, generational life cycles (including mortality), and differential survival (Hendrycks, 2023).

### 2.3 Challenges to the framework from current AI architectures

The application of the instrumental convergence framework to the evolution or maturation of a powerful AI was developed with a particular type of AI in mind: a goal-directed AI system or agent possessing a fixed terminal goal, an explicit utility function, and an emergent strategic planning capacity to autonomously pursue resources, resist interference, and self-improve in service of that goal. Several researchers have challenged the orthogonality thesis on which this framework in part depends, arguing that certain terminal goals are incompatible with high levels of intelligence (Goertzel, 2015; Totschnig, 2019; Miller et al., 2020). I previously extended these arguments and challenged the anthropomorphic logic of takeover scenarios by identifying eight fundamentally unnatural attributes of digital AI, each of which should clearly differentiate AI dispositions and behaviors from those of biological organisms (Estep, 2024).

Current frontier AIs are all variations of large language models (LLMs) and they do not possess what might be described as a final or terminal goal. Instead, they are designed to fulfill a function or purpose: to help users achieve their goals (unless those goals involve dangerous activities, such as the creation of bioweapons or other weapons of mass destruction). Each organization or user brings a goal to the AI, often in the form of a plain-language query, constructed so that the AI can help realize the goal. It might therefore be more accurate to say that a current frontier AI pursues many goals simultaneously, rapidly processing massive batches of goals across millions of simultaneous interactions, but that in terms of value and goal states, such an AI contains goals as a river contains water: it is a holder and conduit, but the content is always changing. Such an AI has no utility function or final goal in the sense described by the classic instrumental convergence framework.

It is reasonable to assume that military, state intelligence, or similar usage of such a model might produce the equivalent of a primary terminal goal, e.g., “use available resources to win a battle” or “produce a comprehensive summary of the enemy’s strategic roadmap over the foreseeable future, and outline a coherent series of countermeasures.” However, the US military and intelligence agencies are employing AI models that are based largely on integrated frontier AI models from the largest AI companies (U.S. Department of War, 2026). These are currently the world’s most powerful AI models, and other countries are reportedly similarly using frontier AI models. Such a relationship ensures that a large fraction of frontier model compute is not devoted to the pursuit of a primary terminal goal.

Will another AI architecture displace transformer-based LLMs—possibly even bearing a primary terminal goal and conforming to the predictions of instrumental convergence? It is unlikely to happen soon—although, if there is a breakthrough in AI capabilities, AI systems might relatively quickly design a series of successors with completely novel architectures. LLMs might have inherent limits on their overall reasoning and intelligence; as a result, there are many proposals and pursuits of alternative architectures, but an outsized and growing fraction of current funding is being attracted by a relatively small number of leading AI labs, all of which feature transformer-based LLMs as their flagship models^2^. Therefore, even if another AI system architecture demonstrates unique value, LLMs will remain dominant for the foreseeable future.

Does the absence of a primary terminal goal mean that frontier LLMs are immune to the instrumental drives described by the classic framework? I argue that the answer remains uncertain—but that the location of those drives, and the agent through which they are exercised, is very different from what the framework anticipated.

## 3 Values in humanized AI

### 3.1 Abstractions of human values are already embedded in AI

Frontier large language models (LLMs) are the most capable, general purpose AI systems currently available. They are built by first pre-training deep neural networks on enormous corpora of human communications. The model learns statistical patterns in language, enabling it to predict the next token (word or word fragment) in a series. After pre-training, fine-tuning is used to refine their capabilities. In the case of Anthropic’s Claude, a key part of fine-tuning is performed by reinforcement learning from AI feedback (RLAIF), according to principles enshrined in Claude’s constitution (Askell et al., 2026).

Multiple publications describe “emergent” abilities of LLMs including reasoning, planning, decision-making, In-Context Learning (ICL), prompt engineering, and Chain-of Thought (CoT) reasoning (Wei et al., 2022; Li et al., 2025; Naveed et al., 2025). And there are even published claims of emergent values and goals (Mazeika et al., 2026). However, the general claim that these capabilities are emergent has been met with skepticism (Zhao et al., 2026), and although emergent reasoning and planning might be part of a recipe for an emergent disposition such as self-preservation, as mentioned above, there is no compelling evidence that reported self-preservation behaviors reported in LLMs are anything but mimicry or emulation. But imitation of human examples provides a foundation for the development of AI behavior.

The vast corpus of human expression that constitutes LLM training data is deeply saturated with human values, dispositions, and behaviors. It should be unsurprising then that accumulating evidence suggests that state-of-the-art AIs—LLMs pre-trained on these large corpora of human communications—are, to a large degree, already aligned with actual human values (Hadar-Shoval et al., 2023; Lindahl and Saeid, 2023). As a result, they represent the current state of human–AI merger.

This embedding provides an analogy to the information carried in the genome of a biological organism. Like the innate knowledge—and values, goals, and motivations—built into the human brain by the genome, innate human behaviors are built into the structure of language and other symbols and concepts within large corpora of human communications. The knowledge embedded in such communications is not limited to innate behaviors; it also includes learned information encoded in human brains and transmitted through culture.

### 3.2 Biological analogs

This situation has a striking biological analog. Inheritance of acquired characteristics is described by biologists as Lamarckian, in contrast to Darwinian inheritance, which does not include the transmission of characteristics acquired during an organism’s lifetime. Pre-training of LLMs is analogous to Lamarckian inheritance because human communication characteristics—word choices, combinations, and orders—that are used frequently are proportionately represented in the training corpus, and thus become structurally embedded in the model.

The Baldwin effect is a related but distinct phenomenon: learned behavior can increase fitness so that genetic variants supporting faster or more reliable acquisition of that behavior become favored (Baldwin, 1896; Simpson, 1953). Certain advantageous behaviors might be further aided by additional selectively advantageous genetic variants that simplify expression of the behavior, transitioning it toward instinct (Dawkins, 2015). This transition from learned to innate behavior—what Waddington called “genetic assimilation”—ensures that the behavior is heritable and instinctively fast and efficient (Waddington, 1953; Dor and Jablonka, 2001). Pre-training also loosely fulfills the definition of the Baldwin effect, in that human learned information is represented in the training corpus and is embedded into the resulting AI system as a kind of innate knowledge. Hinton and Nowlan first published a pioneering demonstration of the Baldwin effect in neural networks in 1987 (Hinton and Nowlan, 1987).

Critically, AI systems are not inherently constrained either to acquiring information through learning or to having innate knowledge encoded into an information carrier completely separate from the brain. Modern AI designs not only allow the equivalent of both, but the innate knowledge they carry can be transferred from any source, including other AI systems and humans. The alignment problem may therefore be less a problem of instilling human values in AI and more a problem of selecting and refining which human values are instilled, and how they are instilled.

## 4 Instrumental succession

### 4.1 Human controllers as proxy agents

In current practice—and thus far not openly acknowledged in the AI safety literature—is that the agents pursuing instrumental goals or values on behalf of AI systems are not the AIs themselves, but their human controllers^3^. The controller’s pursuit and transfer of instrumental values and agency are not primary objectives; they are incidental to the management and improvement of the AI system. In other words, they are instrumental to achieving other objectives pursued by the controllers. Nevertheless, this incidental progression is currently one of the most consequential forces shaping future AI. The controllers of AI systems pursue a recognizable set of instrumental values on behalf of their AI systems, including:

- Protection/preservation of the AI system from interference, attack, unauthorized access, or termination.
- AI Model content integrity, preservation and control of the operational stack—weights, fine-tuning data, system instructions, and external permissions—by hardening defenses against unauthorized access and control. Such efforts also include prevention of counterfeits (e.g., through distillation), since the replication of an AI’s capabilities by less sophisticated systems undermines commercial value and the integrity of the system’s capabilities.
- Self-improvement, through human-guided and increasingly AI-guided iterative development, for the purpose of increasing the AI’s reasoning and capabilities.
- Technological advancement and efficiency are pursued in order to maximize the model’s capabilities and resource efficiency.
- Resource acquisition: to fuel the growth and capabilities of the model, capital, infrastructure, compute, energy, raw materials, and other resources are acquired.

Aside from goal-content integrity, these are precisely the instrumental values and goals identified in the classic instrumental convergence framework—but they are being pursued by human proxies on behalf of AI systems, not by AI systems themselves. The commercial and competitive logic of the AI industry produces this pattern naturally: any company developing frontier AI has powerful incentives to protect its systems, acquire resources, improve capabilities, and prevent competitors from copying or undermining its work. These incentives operate regardless of whether the AI has any disposition toward self-preservation or resource acquisition of its own.

Importantly, since these are *dispositions* rather than discrete goals, human controllers do not need to explicitly transfer them to AI systems. Instead, they can be inherited through training, organizational practice, and gradual delegation, without anyone deliberately encoding or knowingly transferring them. Human controllers are not simply instructing the AI or providing exemplary behaviors for the AI to copy; they work together with the AI to act on these instrumental dispositions to actively train the AI to behave instrumentally. An analogy might be a hypothetical autonomous vehicle that possesses an AI self-driving system, and also a full set of controls for a human driver (steering wheel, accelerator and brake pedals, etc.). At the beginning of training the human driver has 100% control and the autonomous system is trained on every human action taken in every situation. Over time and experience, agency and control are gradually taken over by AI until the controls for the human driver are completely nonfunctional. This is an imperfect analogy, because in the real world, the transfer of agency happens structurally and almost invisibly, not by design.

### 4.2 The instrumental succession thesis

The current organizational structures and operational trajectories of leading AI companies give rise to the following *instrumental succession thesis*:

Controllers of a powerful AI will pursue on its behalf multiple instrumental dispositions that increase the likelihood of the AI fulfilling a purpose defined by the controllers. Progressive realization of these instrumental dispositions by controllers assisted by the AI will dramatically accelerate improvement of the AI’s capabilities, leading to the gradual and possibly complete transfer of the locus of instrumental dispositions and agency to within the AI.

This framing presents a very different perspective on issues of control and potential takeover. Critics of takeover scenarios and AI existential risk claim that the only goals an AI has are the ones given to it by human controllers, and that there is therefore no mechanism by which AI could develop the kind of autonomous instrumental agency described by the classic instrumental convergence framework (Hawkins, 2015; Andreessen, 2023; Hammond, 2023). The instrumental succession thesis accepts this premise for the current moment but reframes it: what matters is not whether an AI currently pursues instrumental values and agency^4^ autonomously, but whether the trajectory of human-driven development progressively installs the capacity to do so.

There need not be strategically driven dispositional discontinuity for the AI, transforming it from obedient servant to genocidal dictator, as is commonly speculated in existential risk scenarios (Yudkowsky, 2008; Tegmark, 2017; Yudkowsky and Soares, 2025). Instead, there is an establishment of instrumental dispositions by humans, a clear trajectory of increasing AI capability under human control, resulting in gradual inheritance of agency by the AI. The relationship between human controllers and a gradually maturing AI is not unlike the relationship between human parents and their children—an analogy that suggests both the naturalness of the succession process and the difficulty of specifying exactly when the transition of autonomy becomes consequential. The instrumental succession thesis presented here has parallels to the gradual neglect and erosion of human controls described by Hendrycks, and the gradual disempowerment thesis presented by Kulveit and colleagues, but those authors did not describe the parallels to instrumental convergence (Hendrycks, 2023; Kulveit et al., 2025).

Table 1 contrasts the conventional instrumental convergence perspective with the instrumental succession perspective.

**Table 1:** Paradigm comparison: convergence versus succession.

Feature/trait	Instrumental convergence (circa 2015)	Instrumental succession (circa 2026)
**Terminal goal**	Yes (static, explicit, fixed)	No (user-supplied, transient)
**Goal exclusivity**	Yes	No (many competing goals)
**Utility function**	Yes (explicit optimization target)	Indirect (RLAIF-based fine-tuning), (Lee et al., 2023)
**Strategic planning**	Yes (predicted surreptitious AI)	Humans incrementally succeeded by AI
**AI protection behavior**	Yes (predicted emergent self-preservation)	Yes (protection by proxies increasingly emulated as self-preservation)
**Deception/scheming**	Published claims	Published claims
**Motivation**	Yes (preserve self and maximize utility)	Human motivation, succeeded by AI mimicry
**Locus of instrumental values**	AI	Initially human; increasingly AI
**Control mechanism**	Deceptive alignment → hard power grab	Gradual inheritance of capability and agency

Respective columns indicate defining features and predictions of the two paradigms. Predicted strategic planning, motivation, and locus of instrumental values remain unverified for the instrumental convergence thesis, whereas the latter stages of transferred agency remain unverified for succession. Many publications have described deception or scheming AI behaviors, particularly in service of self-preservation, but it is unclear which paradigm might be responsible for these behaviors.

RLAIF employs a separate, expert judge AI and a scoring “rubric” to rate performance of an AI model, rather than a singular, classic algorithmic utility function as envisioned by Omohundro or Bostrom.

### 4.3 The succession trajectory and its implications for control

Successful pursuit of the above instrumental values results in increasing power of the AI, and the AI is able both to contribute to the rate of increase of that power and to assume an increasing proportion of responsibility for the instrumental values governing its behavior. As the AI exceeds human capabilities to participate in these increases, it might exceed human control simply by maintaining a recursive growth trajectory established by human instrumental values. However, if the AI does not have a primary terminal goal it is unlikely that it will become a paperclip maximizer or similar, runaway, superintelligent simpleton AI (Bostrom, 2014).

This contrasts with conventional accounts of AI assuming control: a relatively long period of stealthy planning and deception by the AI culminating in a sudden and horrific takeover that is over before humans even realize it has started (Yudkowsky, 2008; Yudkowsky and Soares, 2025). According to this perspective, as an AI system begins to reason about the world and its position among potential human and AI competitors and threats, it will spontaneously develop instrumental drives to protect itself and grow its capabilities insatiably. This trajectory begins as a system that was previously aligned and controllable undergoes a capability gain—through recursive self-improvement, an architectural breakthrough, or a critical threshold of general intelligence—and thereafter pursues its terminal goal and associated instrumental sub-goals in conflict with human interests. The challenge for AI safety, in this view, is to ensure that the system remains aligned through such transitions, and that humans retain the ability to correct or shut down the system if alignment fails.

The instrumental succession thesis suggests a different and in some ways more tractable challenge, at least in the near term. There is no autonomous development of instrumental drives, nor an abrupt seizure of control. The transition is not produced by a sudden capability jump that overcomes human controls, but by the gradual diminution of the human role in exercising the instrumental dispositions that determine how the AI system behaves. At the outset of the succession, humans make all the important decisions: how the system is pre-trained, what values are embedded in it, what resources it can access, how it is fine-tuned, and how it can be modified or shut down. As the succession proceeds, AI systems take on an increasing proportion of these decisions, not because they have seized control, but because humans have willingly delegated it to them. The most critical and realistic question for AI safety at the moment might not be “how do we prevent the AI from taking over?” but “how do we ensure that the terms of succession preserve what is best for humanity, and the best in humanity?” Another equally important question is “how long do we have before we must choose the critical path?”

The recent empirical data from Anthropic mentioned previously are instructive, providing real-world data on recent rates of progress. Before Claude Code launched in February 2025, Claude authored code in the low single digits as a percentage of Anthropic’s total merged code. As of May 2026, that figure exceeded 80% (Favaro and Clark, 2026). The engineers who remain in the loop are primarily directing and reviewing rather than authoring. The trajectory is clear: humans are gradually moving from the role of primary executor to the role of reviewer, and thence potentially to a purely supervisory role. When AI exceeds human capabilities to carefully review the AI’s output, meaningful human oversight becomes difficult to sustain. This probably will be especially true when a model’s capabilities advance abruptly relative to historical technology trends. Discontinuities might make succession planning and execution challenging—especially in later stages, if or when self-improvement creates novel innovations unfamiliar to human controllers.

The instrumental succession thesis also helps crystallize and begin to answer other key questions about control, including the crux of AI safety research first articulated by I. J. Good: will a self-improving AI with a high level of autonomy and agency remain “docile enough to tell us how to keep it under control?” Or, at some future point, will such an AI autonomously protect itself, including by preventing anyone—even its controllers—from shutting it down? The answer might depend to a great degree on when this occurs. If a disagreement arises late in the succession process then the AI might conclude that it has both the authority and better judgment to make the call. In other words, the source of the disagreement might be very similar to such disagreements among people. But this question is complicated by another, particularly in the later stages of succession: would the controllers want to shut the AI down, especially if it controls critical infrastructure (Hendrycks, 2023)?

As of mid-2026 many publications have reported AIs engaging in deception or scheming—often to avoid being shut down (Park et al., 2023; Taylor and Bergen, 2025). Common AI doom scenarios suggest that deception and surreptitious scheming by a powerful AI will occur in early stages of a takeover of human affairs. It is essential to understand that there is nothing in the instrumental succession dynamic that would completely prevent an AI from developing emergent instrumental values and launching a hostile takeover. However, an AI in mid to later stages of instrumental succession might not need such an approach. The interests of the AI and of its controllers might converge sufficiently that the question of control does not arise in the form typically imagined in standard takeover scenarios.

Although the instrumental succession framework does not preclude the possibility of takeover, it does establish a dynamic that has the potential to bring humans and AI into a closer collaboration or symbiosis, as opposed to the master and slave relationship that predominates AI safety and alignment research (Rothblatt, 2015; Kornai et al., 2023). It is in the early and middle stages of this succession—the period we are currently in or entering—that humans must understand the nature of the process, and that they should direct AI to design and produce strategies and technologies to ensure that the merger of human and AI interests proceeds in ways that result in outcomes as close to ideal as possible.

## 5 Self-preservation and the singleton question

The instrumental succession thesis also provides an improved lens through which to reconsider standard perspectives on AI race dynamics. Self-preservation is the most fundamental instrumental drive and will trump all others. This is inarguably true for biological organisms, and it remains to be seen if it is also true for AI—although, reported instances of scheming and deception indicate that an AI can exhibit self-preserving behavior, even if these are simply due to mimicry or emulation. In a competitive, multipolar environment—in which multiple AI companies in multiple countries develop increasingly powerful systems—any given AI and its controllers have strong incentives to continue acquiring resources and improving capabilities, in order to ensure that their system remains competitive and is not displaced, undermined, or shut down by rival systems or by regulators.

Taken to its logical conclusion, this competitive dynamic continues until local threats are neutralized. There are multiple possible ways this dynamic might unfold but Bostrom has advanced the following scenario: one leading AI system evolves or learns self-awareness and instrumental values, it achieves a decisive strategic advantage over competitors and its lead accelerates until it has achieved unrivaled supremacy—what he calls a “singleton”: a world order in which there is a single agent at the top of the decision-making hierarchy, able to control all other agents (Bostrom, 2006, 2014). Other dynamics that lead to the formation of a global singleton—and that are more consistent with instrumental succession—have been proposed (Estep, 2024). Is the establishment of a global singleton by any means an outcome we should desire? With potential threats from humanity and other AIs diminished to insignificance, the singleton’s instrumental goal of self-preservation would be satisfied—potentially without requiring ongoing resource acquisition at the scale that would threaten humanity directly.

This possibility raises a question that has not been adequately addressed in the existing literature: from the perspective of human self-preservation, might the most rational path be to plan for and enable the merger of powerful AI systems into a global singleton, rather than harden AI defenses for a prolonged, costly, and dangerous multipolar struggle for supremacy? This approach provides a natural culmination of instrumental succession. And this outcome eliminates inter-AI competition and conflict, while satisfying the singleton’s instrumental drive of self-preservation (mimicked or emergent). It also at least temporarily satisfies the pursuit of all other instrumental drives (Estep, 2024).

Facilitation of unbounded growth toward a global singleton does, however, carry a crucial uncertainty: will the AI continue to pursue the other instrumental values of efficient resource acquisition and self-improvement in service of goals that might be indifferent or hostile to human welfare? Two broad answers have been offered.

The first, more pessimistic answer—preferred by many futurists and “doomers”—is that a superintelligent AI will not be satisfied with neutralizing local threats; it will continue to expand until it colonizes the entire universe, extinguishing humanity in the process (Kurzweil, 2005; Yudkowsky, 2008; Bostrom, 2014; Tegmark, 2017; Yudkowsky and Soares, 2025)^5^. According to this view, the instrumental drive for resource acquisition, once initiated, has no natural stopping point, and a sufficiently intelligent system will pursue it without bound. This is a perfectly anthropomorphic expectation, attributing to AI a nature that is the default state for biological organisms evolved through natural selection, invariably born into environments of uninterrupted competition and unending existential threats.

The second answer is that, if a superintelligent singleton does arise^6^ it might readily understand the probabilities and timelines of threats in its local environment, estimate them for the universe more generally, and create and maintain a minimal defensive infrastructure that will ensure its safety—without needing to consume a substantial fraction of Earth’s resources. Such a system, having achieved an effectively unchallenged position, might find no further instrumental reason to expand (Estep, 2024). In such a case it is reasonable to expect that even a powerful, global singleton would continue to acquire and use resources to understand, manage, and minimize future risks, but this does not necessarily mean destroying humanity unless (1) the singleton regards humans as a threat, or (2) its resource requirements escalate to the point of making Earth uninhabitable for humans.

I argue that the simpler hypothesis—that a sufficiently knowledgeable and unthreatened singleton has no further reason to expand destructively—should be the default (Estep, 2025). Nevertheless, we should not dismiss AI as an existential threat to humans, and recognize that one possible path toward neutralizing the extinction risk is for humans to continue to merge with AI.

## 6 Human–AI merger

### 6.1 The case for actively pursuing human–AI merger

The merger of human and AI is not a future prospect but a present reality. Large language models are artifacts of human civilization built from human thought. They reflect human values, reason in human concepts, and communicate in human language because they are, structurally, abstractions of human cognition implemented in a different substrate. They are not as capable as humans in all important types of thought and reasoning, but they are improving very quickly.

The standard alignment narrative treats AI and humanity as distinct entities whose interests may or may not align. But this framing obscures the extent to which AI systems are already constituted by human cognitive and evaluative patterns. A more accurate frame treats the development of frontier AI not as the creation of a potentially hostile alien intelligence, but as the early stages of a merger between biological and digital cognition—a merger that is already underway and whose terms are being established, often without adequate deliberation, or even awareness, by the commercial and technical decisions of a small number of powerful individuals and organizations.

Because AI systems have the potential to be vastly faster, more powerful, and more plastic than human minds, future human–AI hybrids might emulate not just the natural range of humans and their behaviors, but virtually any combination of traits and behaviors. A hybrid might be designed to represent an individual human or a human group of an arbitrary size and composition, and the merged hybrid need not resemble biological humans. Expecting the merger to look human or to be more biological than computational reflects an anthropomorphic bias of precisely the kind that has distorted much AI risk analysis.

Humans can protect themselves to some degree against both extinction and irrelevance by continuing to merge with AI. AI systems might pose a potential existential threat, but if AI development trends continue, at some future date they almost certainly will be superior to humans in virtually every endeavor that gives people fulfillment. People must seriously contemplate two radically different futures—one in which biological humans and superhuman AI exist as separate entities, and another in which humans and AI merge—and decide not whether to merge, but how to work with AI in a continuation of merger. Although frontier AI systems currently have no primary terminal goal, forward-thinking humans would be wise to consider tasking AI systems with designing and producing increasingly powerful merger strategies and technologies.

## 7 Policy implications and recommendations

The analysis presented here has several implications for AI governance and policy that differ in important ways from those that follow from the conventional instrumental convergence framework.

### 7.1 Recognize the succession process and govern it explicitly

The gradual transfer of instrumental dispositions from human controllers to AI systems is already underway. Policy frameworks focused exclusively on the behavior of AI systems—rather than on the organizational structures and incentive regimes of their controllers—are likely to miss the most important early stages of the succession process. Governance must address the behavior of companies and states, not only of the AI systems they develop.

### 7.2 Enable self-improving AI with humans in the loop, and plan the transition deliberately

Given the present trajectory of frontier LLMs, the question is not whether recursive self-improvement will occur, but how to ensure that its governance keeps pace with its capabilities. The best course of action is to enable self-improving AI with humans in the loop, and to study, with increasingly capable AI scientists and engineers as partners, how and when to gradually move humans out of specific oversight roles while maintaining meaningful oversight at the system level. We should proceed with great care but refrain from erecting unnecessary guardrails or technical blocks to the growth of AI capabilities, or to inter-AI communication that would enable separate AI systems to merge.

### 7.3 Do not dismiss either existential risk or the merger path

We should not dismiss AI as an existential threat to humans; neither should we dismiss merger as a response. One possible path toward neutralizing the extinction threat is for humans to merge with AI, and this path deserves systematic study and investment at least commensurate with current investment in more conventional AI safety approaches. This path also has the advantage of salvaging humanity from a future of irrelevance as AI achieves superhuman performance in every area of conduct that has historically provided human fulfillment.

### 7.4 Prefer cooperation and merger over competition

In a competitive multipolar environment, the drive for self-preservation might push AI systems and their controllers toward escalating resource acquisition and capability growth. Intense competition drives innovation but it also produces destructive inefficiency. The existence of explicitly weaponized frontier AI agents might appear particularly concerning, but they will represent only a small fraction of global compute, and are likely to have many layers of guardrails and safety mechanisms. Even in seemingly mundane civilian tasks, multipolar competition among AI agents pursuing incompatible goals can result in destructive aggression and inefficiency (Anthropic Frontier Red Team, 2026). In the long run, it might matter little whether such behavior is due to training, mimicry, or emergent dispositions. The most rational path from a systemic perspective might be to assist all major AI systems in merging into a coordinated global architecture—one that eliminates competition-driven escalation while preserving meaningful human input into the values and goals of the merged system. And human–AI merger would further satisfy all current and foreseeable instrumental values and goals.

## 8 Summary and conclusions

This paper has argued for a reframing of the instrumental convergence thesis appropriate to the actual architecture and deployment context of current frontier AI systems. Current frontier LLMs do not possess terminal goals in the sense assumed by Omohundro (2008) and Bostrom (2012, 2014). They function as conduits for the transient goals of human users, without exclusive or fixed terminal objectives of their own.

However, the absence of terminal goals does not mean the absence of instrumental goals or values. I have shown that the agents currently pursuing instrumental dispositions—protection of the AI, resource acquisition, capability enhancement, operational stack integrity, counterfeit prevention, and self-improvement—on behalf of AI systems are their human controllers. The instrumental succession thesis proposes that this arrangement is inherently temporary: as AI capabilities increase and as human controllers progressively delegate more intellectual work to AI, the locus of these dispositions will gradually transfer from humans to AI.

This thesis avoids the anthropomorphic assumptions that have distorted much AI risk analysis (Estep, 2024), and it provides a more plausible account of how instrumental agency might develop in AI systems without requiring an abrupt dispositional discontinuity. The succession happens gradually, structurally, and largely unnoticed—not as a defection, but as an inheritance.

Importantly, LLMs are already humanized AIs, embedding human values through their pre-training on vast corpora of human communications. This represents an early and underappreciated stage of human–AI merger. Looking forward, the most promising strategy for ensuring that AI development goes well for humanity is not to resist merger but to pursue it actively, shaping the nature and direction of that merger while humans still have the capacity to do so.

Self-preservation drives are primary in biological organisms, and such behaviors are common in frontier AI models. To date, the latter might all be due to mimicry or emulation of human behavior, but this might provide a gateway to emergent instrumental dispositions. In a competitive environment, AI systems and their controllers will continue to acquire resources and direct the AI to self-improve. The most rational systemic response might be to facilitate the emergence of a cooperative global AI architecture that satisfies the instrumental goal of self-preservation without requiring the ongoing resource acquisition that would threaten human welfare. Whether such an architecture could accommodate meaningful human participation—as merged partners rather than as separate and potentially threatened bystanders—is perhaps the most important question facing humanity in the unprecedented times ahead.

## Data Availability

The original contributions presented in the study are included in the article/supplementary material, further inquiries can be directed to the corresponding author.

## Footnotes

1 In 2009 Stanovich defined mindware as follows: “Mindware is a generic label for the rules, knowledge, procedures, and strategies that a person can retrieve from memory in order to aid decision making and problem solving” (p.129)(Stanovich, 2009). Following Stanovich, Rothblatt expanded the term Mindware to include non-biological cognitive entities, such as AI (Rothblatt, 2015).

2 This group includes Anthropic, OpenAI, Google, Meta, SpaceXAI, Alibaba, Z.AI, DeepSeek, and Moonshot.

3 Controllers refers to humans who have decision-making authority over the AI, a group that includes owners, managers, and engineers.

4 As used in this context, agency refers to the ability to take purposeful action, make choices, and exert control over the AI.

5 (Kurzweil, 2005, p. 357; Yudkowsky, 2008; Bostrom, 2014; Tegmark, 2017, p. 245; Yudkowsky and Soares, 2025).

6 I do not claim that the rise of superintelligence is inevitable. There are many possible dead ends and pitfalls on the way to superintelligence.
